# SARS-CoV-2 Reinfection Risk in Persons with HIV, Chicago, Illinois, USA, 2020–2022

**DOI:** 10.3201/eid2911.230577

**Published:** 2023-11

**Authors:** Richard A. Teran, Alexandra Gagner, Stephanie Gretsch, Jeff Lauritsen, Daniel Galanto, Kelly Walblay, Peter Ruestow, Colin Korban, Massimo Pacilli, David Kern, Stephanie R. Black, Irina Tabidze

**Affiliations:** Centers for Disease Control and Prevention, Atlanta, Georgia, USA (R.A. Teran);; Chicago Department of Public Health, Chicago, Illinois, USA (R.A. Teran, A. Gagner, S. Gretsch, J. Lauritsen, D. Galanto, K. Walblay, P. Ruestow, C. Korban, M. Pacilli, D. Kern, S.R. Black, I. Tabidze)

**Keywords:** COVID-19, HIV/AIDS, SARS-CoV-2, severe acute respiratory syndrome coronavirus 2, viruses, respiratory infections, zoonoses, PWH, PWOH, reinfection, public health surveillance, Chicago, Illinois, United States

## Abstract

Understanding if persons with HIV (PWH) have a higher risk for SARS-CoV-2 reinfection may help tailor future COVID-19 public health guidance. To determine whether HIV infection was associated with increased risk for SARS-CoV-2 reinfection, we followed adult residents of Chicago, Illinois, USA, with SARS-CoV-2 longitudinally from their first reported infection through May 31, 2022. We matched SARS-CoV-2 laboratory data and COVID-19 vaccine administration data to Chicago’s Enhanced HIV/AIDS Reporting System. Among 453,587 Chicago residents with SARS-CoV-2, a total of 5% experienced a SARS-CoV-2 reinfection, including 192/2,886 (7%) PWH and 23,642/450,701 (5%) persons without HIV. We observed higher SARS-CoV-2 reinfection incidence rates among PWH (66 [95% CI 57–77] cases/1,000 person-years) than PWOH (50 [95% CI 49–51] cases/1,000 person-years). PWH had a higher adjusted rate of SARS-CoV-2 reinfection (1.46, 95% CI 1.27–1.68) than those without HIV. PWH should follow the recommended COVID-19 vaccine schedule, including booster doses.

HIV can compromise the immune system; persons with HIV (PWH), especially those not receiving antiretroviral therapy (ART), might be vulnerable to SARS-CoV-2 infection. Studies have reported an increased risk for SARS-CoV-2 infection among those with immunocompromising conditions (*1*–*3*); older age and underlying health conditions are known to increase the likelihood of severe COVID-19 outcomes (*4*,*5*). Understanding how COVID-19 affects PWH is important because approximately half of PWH are >50 years of age (*6*) and have higher rates of medical comorbidities, compared with persons without HIV (PWOH) (*7*,*8*). Studies have reported conflicting results regarding SARS-CoV-2 infection among PWH: adverse COVID-19–related outcomes among PHW (*9*); no association between HIV status and infection risk (*10*,*11*); and increased rates of postvaccination infections in PWH (*12*,*13*). Repeat SARS-CoV-2 infections among PWH and the risk for SARS-CoV-2 reinfection, compared with PWOH, is less understood.

Recovery from SARS-CoV-2 infection is followed by a period of infection-induced immunity, during which antibody titers persist for several months after infection (*14*–*16*). Epidemiologic evidence indicates that infection-induced immunity alone does not provide full protection against subsequent infections and that protection wanes over time (*17*–*22*). Observational studies (*23*–*26*) also indicate that SARS-CoV-2 reinfections with different strains are possible even when infection-induced immunity is coupled with vaccine-induced immunity; risk is likely compounded in the presence of more transmissible circulating variants (*27*). We sought to determine whether HIV infection was associated with an increased risk for SARS-CoV-2 reinfection among residents of Chicago, Illinois, USA.

## Methods

### Data Sources

We abstracted all variables from public health surveillance and information systems. In Illinois, healthcare providers and laboratories are required to report both SARS-CoV-2 and HIV infections among residents to public health. Most SARS-CoV-2 laboratory test results are submitted electronically by testing laboratories directly into the Illinois National Electronic Disease Surveillance System (I-NEDSS). For this analysis, we defined a SARS-CoV-2 case as a positive SARS-CoV-2 nucleic acid amplification or antigen test result from a respiratory specimen collected from a Chicago resident and reported to a public health entity. We considered multiple positive SARS-CoV-2 test results reported to public health occurring within 90 days as part of the same SARS-CoV-2 case; we used the earliest positive specimen collection date as the index date for the infection episode.

The Enhanced HIV/AIDS Reporting System (eHARS) contains records of Chicago residents who have received an HIV/AIDS diagnosis reported to public health. Medical providers must report all new HIV and AIDS diagnoses; laboratories are required to report all positive HIV diagnostic tests, HIV viral load test results, CD4 test results, and nucleotide sequences from HIV genotypic resistance testing.

COVID-19 vaccine doses administered in the state of Illinois must also be reported to Illinois’ Comprehensive Automated Immunization Registry (I-CARE). I-CARE data include information about the person receiving the vaccine dose, dose administration date, and vaccine manufacturer.

### Surveillance and Information System Matching

The analytic cohort included Chicago residents with a positive SARS-CoV-2 test result reported to I-NEDSS with specimen collection dates on or before March 1, 2022, who were >18 years of age. We followed cohort members until their second positive SARS-CoV-2 test result or their death, or until May 31, 2022. We matched person-level SARS-CoV-2 data from I-NEDSS to person-level I-CARE data to identify all COVID-19 vaccine doses administered to the analytic cohort. We used a modified standardized 12-key hierarchical deterministic algorithm, described previously (*28*). The matching algorithm created 12 variables or keys using a variation of character combinations between first name, last name, and date of birth; key 1 (e.g., full last name + first 6 letters of first name + full date of birth) was the strictest and key 12 the least strict. We created keys for each discrete dataset (i.e., data from I-NEDSS and I-CARE). We compared records across datasets by proceeding chronologically through each key. We flagged matched records and appended them to a separate dataset. We then iteratively compared unmatched records across datasets using key 2 through key 12. We then merged the 12 datasets containing matched records for each key to create a matched dataset.

To identify PWH, we compared the matched dataset to a subset of records from eHARS using a separate 12-key hierarchical deterministic algorithm that also used first name, last name, and date of birth. The eHARS subset included PWH reported in eHARS who were alive as of January 1, 2020, and whose current city of residence was Chicago or whose city of residence at death, HIV diagnosis, AIDS diagnosis, or HIV disease diagnosis was listed as Chicago when current city of residence was unavailable. The final analytic dataset included person-level records containing SARS-CoV-2 data, COVID-19 vaccine data, and HIV data for each person in the analytic cohort.

### Category Definitions

We categorized persons with an HIV infection reported to eHARS before their first reported SARS-CoV-2 positive specimen collection date as PWH. For persons with an initial SARS-CoV-2 positive specimen collection date on or after February 1, 2022, we determined HIV status using eHARS data available as of January 31, 2022, because that was the most recent dataset available for analysis. We categorized persons with no evidence of HIV infection as PWOH. We excluded persons with an HIV diagnosis after their first SARS-CoV-2 infection from the analysis. We defined SARS-CoV-2 reinfection as a SARS-CoV-2 case occurring on or before May 31, 2022, and >90 days from an initial SARS-CoV-2 positive test result.

### Additional Covariates

We obtained age at first infection, sex at birth, race and ethnicity, and address from SARS-CoV-2 data reported to I-NEDSS. If sex at birth and race and ethnicity data were missing, we examined variables in eHARS and I-CARE and reconciled the data in the analytic dataset. We used address to categorize residence into 1 of 7 Chicago regions to account for differences in SARS-CoV-2 incidence and COVID-19 vaccine distribution and uptake across the city. We categorized persons into 4 distinct infection groups (ancestral variant, Alpha and other pre-Delta variants, Delta variant, and Omicron variant) on the basis of the specimen collection date of their initial SARS-CoV-2 infection. We determined thresholds for each group using local and national molecular surveillance data; the starting threshold for a new infection group indicates when 50% of sequenced specimens were attributed to a variant (*29*–*31*).

We evaluated vaccination status at the time of the first SARS-CoV-2 infection, and if applicable, at the time of reinfection. We categorized persons as unvaccinated if they had not received a COVID-19 vaccine dose; partially vaccinated if they received 1 dose of a 2-dose COVID-19 vaccine series; and completed series if they received 2 doses of a 2-dose series or 1 dose of a 1-dose series. We defined an additional vaccine dose as any vaccine dose administered after completing the primary series. We also characterized vaccination status by vaccine manufacturer of the primary series. 

For PWH, we determined years since HIV diagnosis and history of AIDS at initial SARS-CoV-2 infection. We determined history of AIDS on the basis of a clinical opportunistic illness diagnosis or CD4 count <200 cells/mm^3^ before the first SARS-CoV-2 infection (*32*). We identified the laboratory CD4 count and HIV viral load test result preceding the first SARS-CoV-2 infection for each PWH. We categorized most recent CD4 count as <200, 200–349, 350–499, or >500 cells/mm^3^. We used HIV viral load results to describe viral suppression status. We classified viral loads <200 copies/mL as suppressed, consistent with the national HIV surveillance definition of viral suppression (*33*).

The Chicago Department of Public Health Institutional Review Board and the Centers for Disease Control and Prevention reviewed our investigation. We conducted our study in accordance with department policies and applicable federal law (45 C.F.R. part 46.102(l)(2), 21 C.F.R. part 56; 42 U.S.C. §241(d); 5 U.S.C. §552a; 44 U.S.C. §3501 et seq).

### Statistical Analysis

We followed persons in the study from the earliest positive SARS-CoV-2 specimen collection date until the earliest specimen collection date of their second positive SARS-CoV-2 infection occurring >90 days from the first infection; until death; or until May 31, 2022, whichever occurred first. We compared characteristics by HIV status to evaluate differences between the subset of persons who had 1 SARS-CoV-2 infection reported to public health and the subset of persons who had >1 SARS-CoV-2 infection. Among PWH, we compared HIV characteristics by reinfection status.

We calculated SARS-CoV-2 reinfection incidence rates and incidence rate differences per 1,000 person-years by HIV status and for each variant phase and calendar quarter. We used nonparametric survival analyses to estimate cumulative incidence of SARS-CoV-2 reinfection during the observation period. We tested for differences in cumulative incidence curves by HIV status by using Gray’s tests.

To assess the effect of HIV infection on SARS-CoV-2 reinfection during the observation period, we compared crude and adjusted rate ratios using univariable and multivariable Poisson regression models with the log of person-time as the offset term. To account for overdispersion in the Poisson regression model, we calculated robust SEs using generalized estimating equations. We adjusted the model for age, sex at birth, race and ethnicity, region of residence, initial SARS-CoV-2 infection group, vaccination status at the time of first SARS-CoV-2 infection, type of first COVID-19 vaccination dose received at the time of first SARS-CoV-2 infection, and number of doses administered after the first SARS-CoV-2 infection. We used SAS version 9.4 (SAS Institute, Inc., https://www.sas.com) to conduct record matching and analyses. 

## Results

A total of 453,587 Chicago residents meeting the analytic cohort criteria had a positive SARS-CoV-2 test result reported to public health during March 2020–March 2022; those persons contributed a total of 473,513 person-years (median 1.1 person-years; interquartile analysis [IQR] 0.4–1.5 person-years) during the 26-month observation period (Appendix Table). Median age of the analytic cohort was 39 (IQR 28–53) years, and most (71.0% [321,905/453,587]) were unvaccinated at the time of their first SARS-CoV-2 infection. Among the analytic cohort, 2,886 (0.6%) persons had received an HIV diagnosis before their first SARS-CoV-2 infection. In total, 23,834 (5.3%) persons had a second positive SARS-CoV-2 test result reported to public health >90 days after their first infection, indicating a SARS-CoV-2 reinfection. The percentage having a SARS-CoV-2 reinfection was higher among PWH (6.7% [192/2,886]) compared with PWOH (5.2% [23,642/450,701]; p<0.01).

Most (188,155 [41.5%]) persons’ first SARS-CoV-2 infection occurred during March–December 2020, which was the ancestral variant phase of the COVID-19 pandemic (Appendix Table 1). Among 131,682 persons who received COVID-19 vaccine before their first SARS-CoV-2 infection, a plurality (71,333 [54.2%]) had completed a primary vaccine series from Pfizer-BioNTech (https://www.pfizer.com). Of 23,834 persons who had a SARS-CoV-2 reinfection, 39.6% (9,444/23,834) had completed a primary series but had not received an additional dose at the time of their SARS-CoV-2 reinfection. Among persons who experienced a SARS-CoV-2 reinfection, PWH were older (median 43 years of age, 95% CI 32–57 years of age) than PWOH (median 36 years, 95% CI 27–49 years of age; p<0.01). PWH were more likely than PWOH to be male (79.3% vs. 40.9%; p<0.01) and non-Hispanic or Latino Black or African American (53.7% vs. 27.0%; p<0.01), and to have completed a primary COVID-19 vaccination series plus additional dose (31.8% vs. 22.1%; p<0.01). Also, among persons with a SARS-CoV-2 reinfection, PWH were less likely to be unvaccinated at the time of their first infection, compared with PWOH (87.5% vs. 91.0%; p<0.01).

**Table 1:** SARS-CoV-2 reinfection incidence rates, by HIV status, Chicago, Illinois, USA, January 1, 2020–May 31, 2022*

Characteristic	No. (%) events	Incidence, % (95% CI)			Incidence rate difference, %
		Analytic cohort	PWH	PWOH	
Total observation period	23,834 (100)	50 (37–66)	66 (57–77)	50 (49–51)	16
Timing of reinfection, by variant phase					
Ancestral variant, 2020 Jan 1–Dec 31	866 (3.6)	16 (9–26)	30 (16–59)	16 (15–17)	14
Alpha and other pre-Delta variants, 2021 Jan 1–Jul 10	1,030 (4.3)	6 (2–13)	8 (4–16)	6 (5–6)	2
Delta variant, 2021 Jul 11–Dec 18	3,039 (12.8)	11 (5–19)	17 (12–25)	11 (10–11)	6
Omicron variant, 2021 Dec 19–2022 Mar 1	18,899 (79.3)	40 (29–55)	50 (42–60)	40 (39–41)	10
Timing of reinfection, by calendar quarter					
2020					
Apr–Jun	14 (0.1)	2 (0–7)	16 (1–209)	2 (1–3)	14
Jul–Sep	268 (1.1)	12 (6–21)	30 (11–82)	12 (10–13)	18
Oct–Dec	584 (2.5)	11 (5–20)	14 (5–37)	11 (10–12)	3
2021					
Jan–Mar	549 (2.3)	5 (2–12)	5 (1–16)	5 (4–6)	0
Apr–Jun	457 (1.9)	3 (1–8)	4 (1–12)	3 (2–3)	1
Jul–Sep	721 (3.0)	3 (1–9)	9 (5–17)	3 (2–-3)	6
Oct–Dec	9,477 (39.8)	32 (22–45)	42 (33–53)	32 (31–32)	10
2022					
Jan–Mar	7,750 (32.5)	20 (12–31)	27 (21–35)	20 (19–20)	7
Apr–May	4,014 (16.8)	9 (4–17)	9 (5–13)	9 (8–9)	0

*Incidence is given as percentage of cases per 1,000 person-years. PWH, persons with HIV; PWOH, persons without HIV.

Among PWH (n = 2,886), median time from HIV diagnosis to initial SARS-CoV-2 infection was 12.8 (IQR 6.3–19.7) years. Approximately half (1,359 [47.1%]) met criteria for AIDS before their first SARS-CoV-2 infection. More than half (1,513 [52.4%]) had a CD4 count >500 cells/mm^3^, and most (2,213 [76.7%]) had an HIV viral load result <200 copies/mL, indicating viral suppression. HIV history data and HIV laboratory data were not significantly different (p>0.05) when stratified by SARS-CoV-2 reinfection status.

Incidence rate of SARS-CoV-2 reinfection for the analytic cohort was 50 (95% CI 37–66) cases/1,000 person-years (Table 1). Reinfection incidence was higher among PWH (66 [95% CI 57–77] cases/1,000 person-years) than PWOH during the observation period (50 [95% CI 49–51] cases/1,000 person-years). When we examined SARS-CoV-2 reinfections by variant phase and calendar quarter, PWH consistently had a higher incidence rate of SARS-CoV-2 reinfection, compared with PWOH. We observed the highest incidence rates for PWH during the omicron variant phase (50 [95% CI 42–60] cases/1,000 person-years); by calendar quarter the incidence rates were highest during October–December 2021 (42 [95% CI 33–53] cases/1,000 person-years). Incidence rate differences between PWH and PWOH varied throughout the observation period; overall, an excess of 16 SARS-CoV-2 reinfections/1,000 person-years among PWH were reported. We observed the highest incidence rate difference during July–September 2020, which was an excess of 18 SARS-CoV-2 reinfections/1,000 person-years among PWH compared with PWOH.

**Figure 1:**
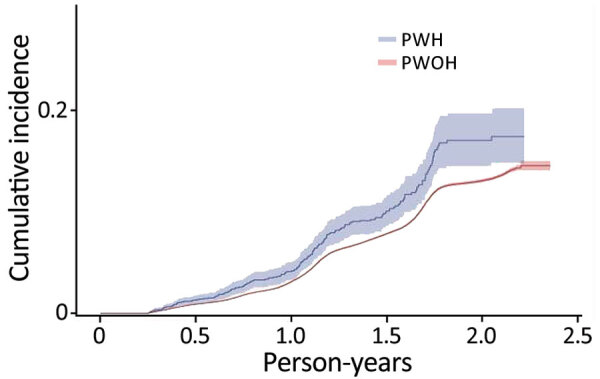
Cumulative incidence (cases/1,000 person-years) of SARS-CoV-2 reinfection by HIV status, Chicago, Illinois, USA, January 1, 2020–May 31, 2022. PWH, persons with HIV; PWOH, persons without HIV.

**Figure 2:**
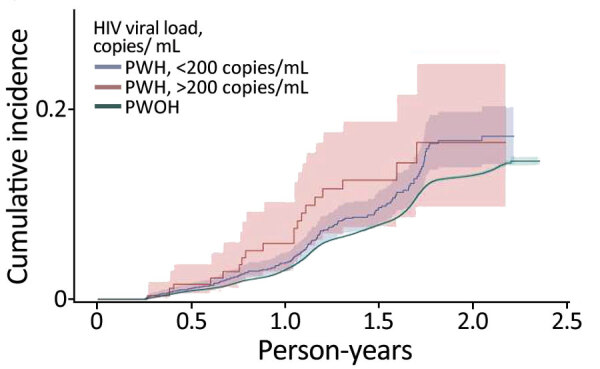
Cumulative incidence (cases/1,000 person-years) of SARS-CoV-2 reinfection by HIV viral suppression status, Chicago, Illinois, USA, January 1, 2020–May 31, 2022. PWH, persons with HIV; PWOH, persons without HIV.

**Figure 3:**
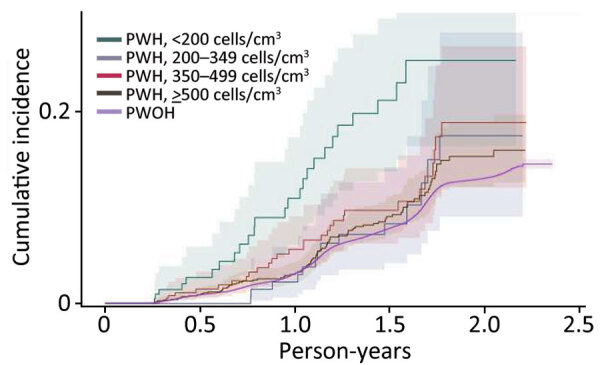
Cumulative incidence (cases/1,000 person-years) of SARS-CoV-2 reinfection by most recent CD4 count, Chicago, Illinois, USA, January 1, 2020–May 31, 2022. PWH, persons with HIV; PWOH, persons without HIV.

**Figure 4:**
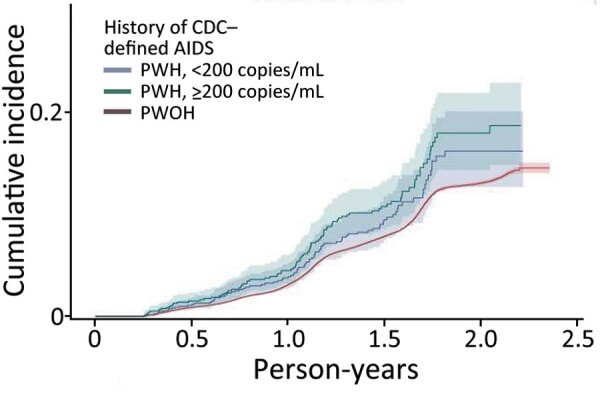
Cumulative incidence (cases/1,000 person-years) of SARS-CoV-2 reinfection by history of AIDS diagnosis, Chicago, Illinois, USA, January 1, 2020–May 31, 2022. CDC, Centers for Disease Control and Prevention; PWH, persons with HIV; PWOH, persons without HIV.

SARS-CoV-2 reinfection cumulative incidence rate (CIR) at 1 year after an initial infection was 3.1% (95% CI 3.0%–3.2%); at 1.5 years, 7.8% (95% CI 7.7%–8.0%); at 2 years, 13.1% (95% CI 12.9%–13.3%), and at 2.3 years, the end of observation period, 14.5% (95% CI 14.0%–15.1%). CIR was higher among PWH than PWOH at all time points (Figure 1). Two years after an initial infection, the SARS-CoV-2 reinfection CIR was 17.0% (95% CI 14.5%–19.7%) among PWH, compared with 13.1% (95% CI 12.9%–13.2%) among PWOH. Persons with HIV who were not virally suppressed had higher SARS-CoV-2 reinfection CIR compared with virally suppressed PWH and PWOH (Figure 2); 1.5 years after an initial SARS-CoV-2 infection, CIR in virally unsuppressed PWH was 12.5% (95% CI 7.6%–18.8%), in virally suppressed PWH was 9.7% (95% CI 8.0%–11.4%), and in PWOH was 7.8% (95% CI 7.7%–7.9%). Similarly, PWH whose most recent CD4 count was <200 cells/mm^3^ had higher SARS-CoV-2 reinfection CIR (Figure 3). CIR at 1.5 years after initial infection among PWH with CD4 count <200 was 21.2% (95% CI 13.7%–29.7%), among PWH with CD4 of 200–349 was 8.3% (95% CI 4.2%–14.2%), among PWH with CD4 of 350–499 was 9.7% (95% CI 6.2%–14.1%), and among PWH with CD4 ≥500 was 9.1% (95% CI 7.2%–11.2%). CIR in PWOH was 7.8% (95% CI 7.7%–7.9%). PWH who had a history of AIDS before their first SARS-CoV-2 infection also had a higher SARS-CoV-2 reinfection CIR (10.8% [95% CI 8.6%–13.2%]) at 1.5 years after initial infection, compared with PWH who had no history of AIDS (9.5% [95% CI 7.5%–11.7%]) and PWOH (7.8% [95% CI 7.7%–7.9%]) (Figure 4).

In unadjusted analyses, compared with PWOH, PWH had a higher rate (rate ratio 1.32 [95% CI 1.15–1.51]) of SARS-CoV-2 reinfection during the observation period (Table 2). After we adjusted for sociodemographic factors, region of residence, initial SARS-CoV-2 infection timing, vaccination status, vaccination type, and number of doses administered after initial SARS-CoV-2 infection, PWH had a higher rate (adjusted rate ratio 1.46 [95% CI 1.27–1.68]) of SARS-CoV-2 reinfection during the observation period compared with PWOH.

**Table 2:** Effect of HIV status on SARS-CoV-2 reinfection, Chicago, Illinois, USA, January 1, 2020–May 31, 2022*

HIV status at first SARS-CoV-2 infection	Total no. (%) persons, n = 453,587 (%)	Total no. (%) events, n = 23,834	Unadjusted rate ratio (95% CI)	p value	Adjusted rate ratio (95% CI)†	p value
PWH	2,886 (0.6)	192 (0.8)	**1.32 (1.15–1.51)**	**<0.0001**	**1.46 (1.27–1.68)**	**<0.0001**
PWOH	450,701 (99.4)	23,642 (99.2)	Reference		Reference	

*Estimates describe exponentiated estimates from a Poisson regression model using the logarithm of person-time as the offset term. Bold text indicates statistical significance at p<0.05. PWH, persons with HIV; PWOH, persons without HIV. †Multivariable Poisson regression model controlled for the following covariates: age, sex at birth, ethnicity and race, region of residence, initial SARS-CoV-2 infection group, vaccination status at the time of first SARS-CoV-2 infection, vaccination type, and number of doses administered after the first SARS-CoV-2 infection.

## Discussion

This population-level analysis of matched records from different public health surveillance and information systems revealed that, among adult Chicago residents who had a reported positive SARS-CoV-2 infection, 5.3% experienced a SARS-CoV-2 reinfection during the observation period. However, incidence of SARS-CoV-2 reinfection was consistently higher among PWH than PWOH. Incidence rate differences fluctuated; differences were greater in SARS-CoV-2 reinfection incidence between PWH and PWOH occurring during periods of high citywide case rates. Moreover, after adjustment for demographic factors, residence, and COVID-19 vaccination, PWH were found to experience a higher rate of SARS-CoV-2 reinfection than were PWOH.

National and local surveillance data distinguishing SARS-CoV-2 reinfections from previous SARS-CoV-2 infections are limited. However, the proportion of persons who experienced a SARS-CoV-2 reinfection in Chicago is consistent with surveillance data from New York state, which reported 5.9% of residents have had a SARS-CoV-2 reinfection (*34*). Compared with observational studies reporting population-level estimates of SARS-CoV-2 reinfections in other US jurisdictions and in countries in Europe, our analysis identified higher reinfection incidence rates than previously reported (*35*–*37*). However, those differences might be attributable to methodological distinctions across studies, including a longer observation period through the Omicron variant phase of the COVID-19 pandemic. Our study also found that reinfection risk increases over time, which is consistent with different studies reporting SARS-CoV-2 infection-induced and vaccine-induced immunity waning over time and immune evasion (*18*–*21*).

Our analysis found that SARS-CoV-2 reinfection cumulative incidence rates were higher among PWH than PWOH irrespective of most recent CD4 and viral load laboratory results. This finding indicates that even persons with well-controlled HIV infection might have a higher risk for SARS-CoV-2 reinfection compared with PWOH. We observed the highest cumulative incidence rates among PWH with a most recent HIV viral load laboratory result >200 copies/mL and CD4 laboratory result <200 cells/mm^3^, indicating that PWH with laboratory evidence consistent with uncontrolled HIV are at greatest risk for multiple SARS-CoV-2 infections. Similarly, PWH with a history of AIDS had a higher cumulative risk for reinfection, followed by PWH without AIDS; PWOH had the lowest cumulative incidence across the 3 strata. Persons who are immunocompromised are at increased risk for severe COVID-19 illness and death; their immune response to COVID-19 vaccination may not be as strong as in persons who are not immunocompromised. Guidance from the Centers for Disease Control and Prevention recommends that persons with advanced or untreated HIV receive an additional COVID-19 vaccine dose to complete a primary series and receive a booster (*38*,*39*). However, our analysis also indicated that PWH with high CD4 counts, no evidence of a prior AIDS diagnosis, and viral suppression are also at higher risk for SARS-CoV-2 reinfection than PWOH. Additional studies are needed to understand if those with well-controlled HIV also require additional COVID-19 vaccine doses.

Multiple studies have shown that persons who receive COVID-19 vaccine after a SARS-CoV-2 infection have a lower risk for reinfection than do unvaccinated persons (*40*–*42*). Most persons in the analytic cohort, regardless of HIV status, were unvaccinated at the time of their first SARS-CoV-2 infection. Most of those infections occurred before COVID-19 vaccine was available. Among persons with a SARS-CoV-2 reinfection, 38.2% (9,039/23,642) of PWOH and 31.8% (61/192) of PWH had either not received any COVID-19 vaccine or had not completed a primary series. All persons, especially PWH, should remain up to date with their COVID-19 vaccines to prevent SARS-CoV-2 reinfections or adverse COVID-19 outcomes.

The first limitation of this study is that we were unable to account for testing practices or healthcare-seeking behaviors in our analysis because surveillance systems do not capture reason for testing. Repeat SARS-CoV-2 infections are more likely to be detected among persons who regularly have access to and participate in SARS-CoV-2 testing, leading to differential SARS-CoV-2 reinfection detection rates among PWH and PWOH. Second, availability of SARS-CoV-2 laboratory and at-home testing evolved over time throughout the observation period. Limited access to testing may lead to unconfirmed infections. Similarly, national distribution of at-home antigen tests starting in January 2022 likely led to underestimation of case counts, because those test results are typically not reported to public health. Therefore, our analysis likely underestimates the true number of SARS-CoV-2 reinfections. Third, our person-time calculations only considered reported positive SARS-CoV-2 test results, death, or the end of the observation period. We were unable to censor persons if they moved out of Chicago or had positive test results not reported to their local public health jurisdiction. Therefore, our incidence rate estimates are likely underestimates because some persons may have contributed less person-time. Fourth, we were unable to evaluate and adjust for comorbidities and ART use in our analyses because that type of clinical information is not reported to public health through traditional electronic laboratory reporting. Data abstracted from medical records might more fully characterize whether comorbidities influence the association between HIV and SARS-CoV-2 reinfection. Last, this analysis assumed all positive test results separated by >90 days were distinct SARS-CoV-2 infection episodes, consistent with the 2021 COVID-19 case definition (*43*). Case reports have documented persistent shedding after a single infection spanning >90 days among persons who are immunocompromised (*44*–*46*), and SARS-CoV-2 reinfections occurring <90 days from a prior infection (*47*,*48*). Because of limited national SARS-CoV-2 whole-genome sequencing capacity and inadequate specimen storage capacity at laboratories, local public health departments are rarely able to verify SARS-CoV-2 reinfections by comparing lineage results from respiratory specimens collected from multiple infection episodes.

All persons, including PWH, should stay up to date with recommended COVID-19 vaccines, including bivalent booster doses (*49*). Evaluating the association between HIV infection and SARS-CoV-2 reinfections using surveillance data can help strengthen public health recommendations including the need for extra doses as part of a primary series, booster doses of vaccine, and optimized ART in PWH. Tailored guidance and prevention messaging for PWH can help reduce the elevated risk we identified in this analysis and limit continued SARS-CoV-2 transmission. As the COVID-19 pandemic persists, local health jurisdictions can leverage data access and link records across surveillance datasets to monitor SARS-CoV-2 infections and other outcomes among vulnerable populations, including PWH.

Appendix Additional information about SARS-CoV-2 reinfection risk in persons living with HIV, Chicago, Illinois, USA, 2020–2022.
