# Supplementary material for: SARS-CoV-2 Reinfection Risk in Persons with HIV, Chicago, Illinois, USA, 2020–2022
Source: Emerg Infect Dis. 2023 Nov;29(11):2257–65. doi: 10.3201/eid2911.230577 (PMC10617359; doi:10.3201/eid2911.230577)
Supplement: Appendix — Additional information about SARS-CoV-2 reinfection risk in persons living with HIV, Chicago, Illinois, USA, 2020–2022. [file 23-0577-Techapp-s1.pdf]

Article DOI: http://doi.org/10.3201/eid2911.230577

EID cannot ensure accessibility for supplementary materials supplied by authors. Readers who have difficulty accessing supplementary content should contact the authors for assistance.

SARS-CoV-2 Reinfection Risk in Persons with HIV, Chicago, Illinois, USA, 2020–2022

Appendix

Appendix Table. Characteristics of adult Chicago residents with a SARS-CoV-2 infection reported to public health (N = 453 587) by reinfection and HIV status — Chicago, Illinois, January 1, 2020–May 31, 2022*

		People with one SARS-CoV-2 infection N = 429,753		People with a SARS-CoV-2 reinfection (two SARS-CoV-2 infections) N = 23,834	
Characteristics (no. with available information)	Overall N = 453,587 No. (%)	People with HIV N = 2,694 No. (%)	People without HIV, N = 427,059 No. (%)	People with HIV N = 192 No. (%)	People without HIV N = 23,642 No. (%)
Age at initial SARS-CoV-2 infection, years, median (IQR)*, †	39 (28–53)	47 (35–57)	39 (28–53)	43 (32–57)	36 (27–49)
Age at initial SARS-CoV-2 infection*, † (y)					
18–29	128,169 (28.3)	319 (11.8)	120,130 (28.1)	28 (14.6)	7,692 (32.5)
30–39	107,136 (23.6)	619 (23.0)	100,464 (23.5)	53 (27.6)	6,000 (25.4)
40–49	78,827 (17.4)	581 (21.6)	74,116 (17.4)	37 (19.3)	4,093 (17.3)
50–59	63,804 (14.1)	672 (24.9)	60,209 (14.1)	39 (20.3)	2,884 (12.2)
60–69	42,319 (9.3)	400 (14.9)	40,184 (9.4)	28 (14.6)	1,707 (7.2)
70–79	20,775 (4.6)	94 (3.5)	19,883 (4.7)	6 (3.1)	792 (3.3)
≥80	12,557 (2.8)	9 (0.3)	12,073 (2.8)	1 (0.5)	474 (2.0)
Sex at birth*, †					
Female	240,525 (53.0)	554 (20.6)	225,963 (52.9)	39 (20.3)	13,969 (59.1)
Male	211,512 (46.6)	2,135 (79.3)	199,562 (46.7)	153 (79.7)	9,662 (40.9)
Unknown	1,550 (0.3)	5 (0.2)	1,534 (0.4)	0 (0.0)	11 (0.0)
Race and ethnicity *, †					
Hispanic or Latino	127,307 (28.1)	514 (19.1)	119,263 (27.9)	38 (19.8)	7,492 (31.7)
Non-Hispanic or Latino Asian	17,867 (3.9)	52 (1.9)	16,977 (4.0)	3 (1.6)	835 (3.5)
Non-Hispanic or Latino Black or African American	102,284 (22.6)	1,327 (49.3)	94,482 (22.1)	103 (53.7)	6,372 (27.0)
Non-Hispanic or Latino White	120,939 (26.7)	527 (19.6)	113,506 (26.6)	43 (22.4)	6,863 (29.0)
Non-Hispanic or Latino Other ‡	23,727 (5.2)	87 (3.2)	22,704 (5.3)	4 (2.1)	932 (3.9)
Unknown	61,463 (13.6)	187 (6.9)	60,127 (14.1)	1 (0.5)	1,148 (4.9)
City Region of Residence *, †					
Central	27,823 (6.1)	75 (2.8)	26,381 (6.2)	4 (2.1)	1,363 (5.8)
Far South	46,621 (10.3)	308 (11.4)	43,829 (10.3)	21 (10.9)	2,463 (10.4)
North	80,081 (17.7)	747 (27.7)	74,952 (17.6)	53 (27.6)	4,329 (18.3)

Characteristics (no. with available information)	Overall N = 453,587 No. (%)	People with one SARS-CoV-2 infection N = 429,753		People with a SARS-CoV-2 reinfection (two SARS-CoV-2 infections) N = 23,834	
		People with HIV N = 2,694 No. (%)	People without HIV, N = 427,059 No. (%)	People with HIV N = 192 No. (%)	People without HIV N = 23,642 No. (%)
Northwest	104,184 (23.0)	260 (9.7)	98,442 (23.1)	24 (12.5)	5,458 (23.1)
South	38,851 (8.6)	497 (18.5)	36,190 (8.5)	28 (14.6)	2,136 (9.0)
Southwest	76,368 (16.8)	307 (11.4)	72,253 (16.9)	20 (10.4)	3,788 (16.0)
West	76,129 (16.8)	481 (17.9)	71,574 (16.8)	42 (21.9)	4,032 (17.1)
Unknown	3,530 (0.8)	19 (0.7)	3,438 (0.8)	0 (0.0)	73 (0.3)
Vaccination status at time of first SARS-CoV-2 infection *					
Completed primary series plus additional dose(s)	35,624 (7.9)	364 (13.5)	34,925 (8.2)	3 (1.6)	332 (1.4)
Completed primary series no additional dose(s)	80,944 (17.9)	568 (21.1)	79,161 (18.5)	13 (6.8)	1,202 (5.1)
Partially vaccinated	15,114 (3.3)	124 (4.6)	14,384 (3.4)	8 (4.2)	598 (2.5)
Unvaccinated	321,905 (71.0)	1,638 (60.8)	298,589 (69.9)	168 (87.5)	21,510 (91.0)
Vaccination status at time of first SARS-CoV-2 infection, by vaccine type*					
Pfizer-BioNTech (completed primary series)	49,538 (10.9)	299 (11.1)	48,532 (11.4)	6 (3.1)	701 (3.0)
Pfizer-BioNTech (completed primary series with additional dose §)	21,795 (4.8)	179 (6.6)	21,432 (5.0)	1 (0.5)	183 (0.8)
Moderna (completed primary series)	24,565 (5.4)	215 (8.0)	23,999 (5.6)	5 (2.6)	346 (1.5)
Moderna (completed primary series with additional dose §)	12,027 (2.7)	162 (6.0)	11,728 (2.8)	2 (1.0)	135 (0.6)
Janssen (completed primary series)	6,401 (1.4)	49 (1.8)	6,198 (1.5)	2 (1.0)	152 (0.6)
Janssen (completed primary series with additional dose §)	1,637 (0.4)	18 (0.7)	1,607 (0.4)	0 (0)	12 (0.1)
Completed primary series – mix or other products ¶	440 (0.1)	5 (0.2)	432 (0.1)	0 (0)	3 (0)
Completed primary series – mix or other products ¶ with additional dose	165 (0)	5 (0.2)	158 (0)	0 (0)	2 (0)
Vaccination status at time of SARS-CoV-2 reinfection, n = 23,834 †					
Completed primary series plus additional dose	5,290 (22.2)	NA	NA	61 (31.8)	5,229 (22.1)
Completed primary series no additional dose	9,444 (39.6)	NA	NA	70 (36.5)	9,374 (39.7)
Partially vaccinated	106 (0.4)	NA	NA	3 (1.6)	103 (0.4)
Unvaccinated	8,994 (37.7)	NA	NA	58 (30.2)	8,936 (37.8)
Vaccination status at time of SARS-CoV-2 reinfection, by vaccine type †					
Pfizer-BioNTech (completed primary series)	5,409 (22.7)	NA	NA	27 (14.1)	5,382 (22.8)
Pfizer-BioNTech (completed primary series with additional dose §)	2,847 (11.9)	NA	NA	23 (12.0)	2,847 (12.0)
Moderna (completed primary series)	3,263 (13.7)	NA	NA	36 (18.8)	3,227 (13.7)
Moderna (completed primary series with additional dose *)	2,194 (9.2)	NA	NA	33 (17.2)	2,161 (9.1)
Janssen (completed primary series)	713 (3.0)	NA	NA	6 (3.1)	707 (3.0)
Janssen (completed primary series with additional dose §)	227 (1.0)	NA	NA	5 (2.6)	222 (0.9)
Completed primary series – mix or other products ¶	59 (0.2)	NA	NA	1 (0.5)	58 (0.2)
Completed primary series – mix or other products with additional dose ¶	22 (0.1)	NA	NA	0 (0.0)	22 (0.1)
Number of COVID-19 vaccine doses administered after first SARS-CoV-2 infection *, †					
No doses	221,887 (48.9)	1,069 (39.7)	211,791 (49.6)	56 (29.2)	8,971 (38.0)
One dose	64,787 (14.3)	526 (19.5)	61,707 (14.5)	29 (15.1)	2,525 (10.7)
Two doses	89,157 (19.7)	453 (16.8)	80,505 (18.9)	62 (32.3)	8,137 (34.4)
Three doses	71,390 (15.7)	498 (18.5)	66,922 (15.7)	44 (22.9)	3,926 (16.6)
Four doses	6,366 (1.4)	148 (5.5)	6,134 (1.4)	1 (0.5)	83 (0.4)
Initial SARS-CoV-2 infection group *					
Ancestral variant (March 1–December 31, 2020)	188,155 (41.5)	1,009 (37.5)	171,068 (40.1)	130 (67.7)	15,948 (67.5)
Alpha and other pre-Delta variants (January 1–July 10, 2021)	65,782 (14.5)	376 (14.0)	60,240 (14.1)	41 (21.4)	5,125 (21.7)
Delta variant (July 11 – December 18, 2021)	69,757 (15.4)	430 (16.0)	67,465 (15.8)	16 (8.3)	1,846 (7.8)

Characteristics (no. with available information)	Overall N = 453,587 No. (%)	People with one SARS-CoV-2 infection N = 429,753		People with a SARS-CoV-2 reinfection (two SARS-CoV-2 infections) N = 23,834	
		People with HIV N = 2,694 No. (%)	People without HIV, N = 427,059 No. (%)	People with HIV N = 192 No. (%)	People without HIV N = 23,642 No. (%)
Omicron variant (December 19, 2021 – March 1, 2022)	129,893 (28.6)	879 (32.6)	128,286 (30.0)	5 (2.6)	723 (3.1)
Time since HIV diagnosis at initial SARS-CoV-2 infection, years, median (IQR), n = 2,886	12.8 (6.3–19.7)	12.7 (6.2–19.6)	NA	13.0 (6.3–20.6)	NA
Time since HIV diagnosis at initial SARS-CoV-2 infection, n = 2,886 (y)					
<1	97 (3.4)	90 (3.3)	NA	7 (3.7)	NA
1–4	464 (16.1)	435 (16.2)	NA	29 (15.1)	NA
5–9	589 (20.4)	558 (20.7)	NA	31 (16.2)	NA
≥10	1,736 (60.2)	1,611 (59.8)	NA	125 (65.1)	NA
HIV infection exposure category, n = 2,886					
Adult MSM	1,713 (59.3)	1,592 (59.1)	NA	121 (63.0)	NA
Adult IDU	115 (4.0)	107 (4.0)	NA	8 (4.2)	NA
Adult heterosexual contact	380 (13.2)	356 (13.2)	NA	24 (12.5)	NA
Adult MSM and IDU	118 (4.1)	109 (4.1)	NA	9 (4.7)	NA
Adult IDU and heterosexual contact	89 (3.1)	81 (3.0)	NA	8 (4.2)	NA
Perinatal exposure	26 (1.0)	22 (0.8)	NA	4 (2.1)	NA
No risk factor reported or identified	445 (15.4)	427 (15.9)	NA	18 (9.4)	NA
History of AIDS at initial SARS-CoV-2 infection #, n = 2,886	1,359 (47.1)	1,260 (46.8)	NA	99 (51.6)	NA
Most recent reported CD4 at initial SARS-CoV-2 infection, cells/mm3, median (IQR), n = 2,780	613.0 (390.5–844.0)	615.0 (393.0–845.0)	NA	591.8 (337.0–824.0)	NA
Most recent reported CD4 at initial SARS-CoV-2 infection, cells/mm3, n = 2,886					
<200	244 (8.5)	219 (8.1)	NA	25 (13.0)	NA
200–349	259 (9.0)	245 (9.1)	NA	14 (7.3)	NA
350–499	380 (13.2)	351 (13.0)	NA	29 (15.1)	NA
≥500	1,513 (52.4)	1,416 (52.6)	NA	97 (50.5)	NA
Missing or CD4 count data >1 y from initial infection**	490 (17.0)	463 (17.2)	NA	27 (14.1)	NA
Most recent reported viral load at initial SARS-CoV-2 infection, copies/mL, n = 2,886					
Virally suppressed (<200)	2,213 (76.7)	2,063 (76.6)	NA	150 (78.1)	NA
Not virally suppressed (≥200)	280 (9.7)	260 (9.7)	NA	20 (10.4)	NA
Missing or viral load data >1 y from initial infection **	393 (13.6)	371 (13.8)	NA	22 (11.5)	NA

Abbreviations: IQR = interquartile range; NA = not applicable; MSM, men who have sex with men; IDU, injection drug use.
* *P* values for characteristic were statistically significantly ($p < 0.05$) different when comparing persons with only one SARS-CoV-2 infection, by HIV status.
† *P* values for characteristic were statistically significantly ($p < 0.05$) different when comparing persons with a SARS-CoV-2 reinfection (two SARS-CoV-2 infections), by HIV status.
‡ Persons with the following races listed in I-NEDSS as American Indian or Alaska Native, Native Hawaiian or other Pacific Islander, other, or multiracial were categorized as Non-Hispanic or Latino Other.
§ Additional dose type does not match the primary series vaccine type in all cases.
¶ Persons received two doses of COVID-19 vaccine. Products were either mixed (e.g., received Pfizer for dose 1 and Moderna for dose 2) or persons received a non-U.S. authorized COVID-19 vaccine for either dose.
# History of AIDS defined as person meeting immunologic or clinical criteria to be categorized as HIV Stage 3.
** HIV CD4 and viral load data were evaluated for all persons with HIV. Last known CD4 and viral load data reported to public health >1 y before the initial SARS-CoV-2 infection was categorized as missing.
